# Evaluation of the quality of life and satisfaction in patients using complete dentures versus mandibular overdentures. Systematic review and meta‐analysis

**DOI:** 10.1002/cre2.347

**Published:** 2020-11-18

**Authors:** Sonia Egido Moreno, Raul Ayuso Montero, Mayra Schemel Suárez, Joan Valls Roca‐Umbert, Keila Izquierdo Gómez, José López López

**Affiliations:** ^1^ Department of Odontoestomatology, Faculty of Medicine and Health Sciences (Dentistry) University of Barcelona, L'Hospitalet de Llobregat Barcelona Spain; ^2^ Oral Health and Masticatory System Group, IDIBELL (Bellvitge Biomedical Research Institute) Barcelona Spain; ^3^ University of Barcelona Dental Hospital (HOUB) University of Barcelona, L'Hospitalet de Llobregat Barcelona Spain

**Keywords:** complete denture, conventional denture, implant retained, overdenture, quality of life, satisfaction

## Abstract

**Background:**

The World Health Organization (WHO) recognizes edentulism as a physical impairment that results in a negative impact in the daily activities.

**Objective:**

The study aimed to compare the satisfaction and the quality of life, in patients treated with implant retained overdentures with two mandibular implants (IOD) against those with mandibular conventional complete dentures (CCD).

**Methods:**

Different search strategies were used to screen for articles in Pubmed/Medline, Cochrane Library and Scielo of the last 17 years (2003–2020). The keywords used were: “quality of life OR satisfaction” AND “complete denture OR conventional denture” AND “overdenture OR implant retained.”

**Results:**

Six articles and two more were added by manual search. The population was 400 in the CCD and 412 for IOD. The mean age was 64.3 ± 6.41 years. The group was comprised of 283 men and 427 women. The scores obtained in the visual analog scale (VAS) before and after the treatment were statistically significant in favor of the IOD for overall satisfaction, (WMD: 12.329; 95% CI: 4.873 to 19.784, *p*‐value = 0.001), comfort, speech and stability. For esthetics and chewing there was non‐significant improvement while hygiene worsened for the IOD. For the comparison after the treatment between both treatment modalities a statistically significant improvement was found in overall satisfaction (WMD: 14.408; 95% CI: 8.589 to 20.226, *p*‐value < 0.001), comfort, speech, chewing and stability in favor of the IOD but not in esthetics or hygiene.

**Conclusions:**

This systematic review and meta‐analysis show the superiority of the IOD, despite is not achieved in all aspects.

## 1 BACKGROUND

The number of edentulous patients is diminishing in all age ranges (Assunção et al., 2010; Kroll et al., 2018), due to the improvement in oral health (L. Zhang et al., 2017). But the number of people that surpass the age of 65 is continuously increasing. Thus, complete tooth loss has decreased by more than 75% for those aged 65 through 74 years over the past five decades in the United States. Improvements in tooth loss measures, such as edentulism and complete tooth retention, have been most significant among the nonpoor, whereas those who are poor have experienced fewer improvements (Dye et al., 2019). In Spain, edentulous people represent about 16.8% to 23.4% of the population (Eustaquio‐Raga et al., 2013). The World Health Organization (WHO) has recognized edentulism as a physical disability (World Health Organization, 2001) that has an effect in the daily activities related to chewing, speech or aesthetic concerns (Dye et al., 2019; Kutkut et al., 2018). Furthermore, the absence of teeth implies nutritional deficits, functional and sensory alterations in the oral mucosa and overall effects in the general health, decreasing the quality of life of the patients (Kroll et al., 2018; Kutkut et al., 2018). The treatment of the complete edentulism includes conventional complete dentures (CCD), implant retained overdentures (IOD) by implants or implant complete fixed dentures (Kutkut et al., 2018).

During a long time, the use of CCD was the only available treatment for the completely edentulous patient (Assunção et al., 2010; Kutkut et al., 2018; Sharma et al., 2017). This treatment has its limitations that will see its usefulness diminish, mainly because the lack of stability due to the initial and progressive bone loss. This lack of stability and retention may cause pain, inflammation, chewing difficulties, speech alterations and nutrition deficits (Assunção et al., 2010; Kroll et al., 2018; Kutkut et al., 2018; L. Zhang et al., 2017). This problem may be reduced with the retention provided by implants in IOD (Visser et al., 2006).

With the previous knowledge, several studies have compared the use of CCD with IOD retained by two implants. These lastly mentioned dentures are considered an alternative that solve many of the limitations encountered by CCD. They enhance the retention and stability; and contribute to the wellbeing of the patients, compared with the CCD, improving their quality of life (Assunção et al., 2007, 2010; Kroll et al., 2018; Kutkut et al., 2018). Therefore, a group of experts established a consensus in the symposium of the McGill University in 2002 (Feine et al., 2002): “The evidence currently available suggests that the restoration of the edentulous mandible with a conventional denture is no longer the most appropriate first choice prosthodontic treatment. There is now overwhelming evidence that a two‐implant overdenture should become the first choice of treatment for the edentulous mandible.” This consensus has increased the number of researchers that study the impact of the IOD, as well as its effects in the quality of life or satisfaction in patients (Feine et al., 2002). Never the less, despite the studies that compare both treatment modalities, the heterogeneity of the methodology in which the results were collected will not allow the formation of definitive conclusions.

In dentistry, the development of tools for the measurement of quality of life is quite recent. In order to consider the treatment options that are available to us the patient perspective is of great interest (Sánchez‐Siles et al., 2018). The tools most frequently used to measure the quality of life are questionnaires using measurement scales such as Likert (Heydecke et al., 2008), visual analog scale (VAS) (Allen et al., 2006) or the Oral Health Impact Profile (OHIP) (Slade & Spencer, 1994).

In this article we will concentrate on those studies that analyze the quality of life and the satisfaction with VAS. This type of scale allowed us to obtain much more subjective information of the patients' experience.

For that reason, we establish our Null Hypothesis (H0): The quality of life and satisfaction of patients that use mandibular IOD retained by two implants is not significantly better than those patients that use mandibular CCD. As our Alternative Hypothesis (H1): The quality of life and satisfaction of patients that use mandibular IOD retained by two implants is significantly better than those patients that use mandibular CCD.

With the objective to answer our hypothesis we undertake a systematic review that answers our PICO question: (Population, Intervention, Comparison, Outcome): Do the mandibular IOD retained by two implants (I) improve the quality of life and satisfaction (O) of patients who are totally edentulous (P) in comparison with those who use mandibular CCD (C)?

## 2 METHODS

### 2.1 Search strategy and article selection

Search was conducted in Pubmed/Medline (NCBI), Cochrane Library and Scielo, of the last 17 years (2003–2020) for articles that compared treatments with maxilar and mandibular CCD versus patients with maxilar CCD and mandibular IOD retained with two implants. The keywords used for this search were: quality of life, satisfaction, complete denture, conventional denture, overdenture and implant retained combined using boolean operators “AND” and “OR”: (quality of life OR satisfaction) AND (complete denture OR conventional denture) AND (overdenture OR implant retained).

With this search strategy, all titles and summaries found were evaluated, duplicate articles were eliminated and the bibliography of the selected studies was reviewed as well as the ones from other reviews in order to include other relevant studies for our own review.

### 2.2 Inclusion/Exclusion criteria

Inclusion: Articles in English and Spanish; Studies that compare treatments of maxilar CCD and IOD versus maxilar and mandibular CCD; Studies that elaborate new CCD and new IOD; Randomized Control Trials (RCT); Studies that use VAS type measurement scales to measure the quality of life and satisfaction; Patients that were previously edentulous (immediate IOD were discarded); Studies that compare the results before and after the treatment established for each group; Studies that use conventional implants.

Exclusion: Studies that use a single implant or more than two implants for the retention of the IOD; Studies where the opposing occlusion is not against CCD; Studies where the CCD is rebased; Use of mini‐implants, short implants or narrow implants; Observational studies, non RCT, reviews or meta‐analysis; and Studies without a control group.

### 2.3 Analysis of satisfaction and quality of life

In our study we will focus on the different parameters studied to value the satisfaction using VAS type scales. A meta‐analysis will be performed to compare the difference between the score the patients reported before the treatment and after the treatment between both groups; as well as the difference only in the post treatment between both groups.

### 2.4 Risk of bias

The clinical trials included for this review were evaluated using the Jadad et al., 1996 scale and the review itself was evaluated using the scale Preferred Reporting Items for Systematic Reviews and Meta‐Analyses (PRISMA) (Moher et al., 2009).

### 2.5 Statistical analysis

The tool used for the statistical analysis was the program OpenMetaAnalyst. The Forest Plots were produced to represent by a graph the difference between the results in satisfaction before and after the prosthetic treatment with a confidence interval (CI) of 95%. The level of significance used was *p*‐value (*p*) = 0.05. The heterogeneity was evaluated with the I^2 test and the Cochrane's Q test.

## 3 RESULTS

**FIGURE 1:**
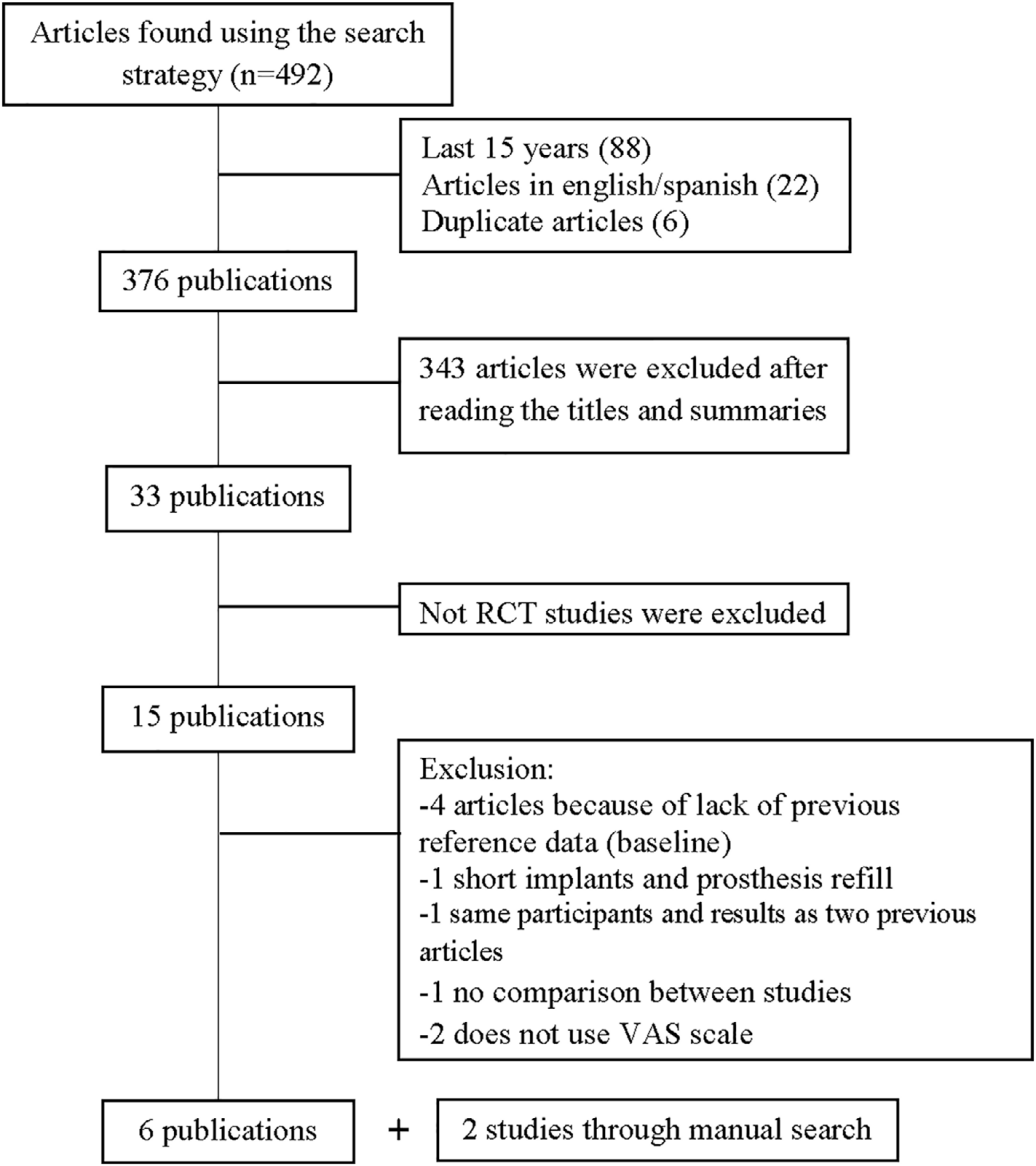
Article selection flowchart

Initially 492 articles were obtained from the search engine Pubmed/Medline (NCBI); no other articles of interest were obtained from any other search engines, by means of the same search strategy, of these articles we concentrated on the ones published over the last 17 years, those written in English or Spanish and after removing the duplicate articles a final total of 376 publications were obtained. After reading the titles and summaries we discarded 343 articles and of the 33 remaining publications 18 studies which were not RCT were eliminated. Of the 15 selected articles, four were excluded because of not providing any reference data; one was excluded given the use of short implants and rebasing the prosthesis; 1 was excluded due to not comparing the results between treatments; 2 more were excluded because they did not use the VAS scale and one article was excluded since it was a recompilation of 2 previously selected studies. At last, six studies were obtained that complied with the inclusion and exclusion criteria (Awad, Lund, Dufresne, & Feine, 2003; Awad, Lund, Shapiro, et al., 2003; Harris et al., 2013; Heydecke et al., 2003; Meijer et al., 2003; Thomason et al., 2003) and two more studies were added with manual search through the bibliography of the selected articles (Pan et al., 2008; Raghoebar et al., 2003). In the search engines Cochrane Library and Scielo no additional articles were obtained (Figure 1).

In Table 1 the methodological quality is represented, through the Jadad scale (Jadad et al., 1996), of the selected articles and after the evaluation of this review through the PRISMA scale (Moher et al., 2009), it fulfilled 22 items.

**TABLE 1:** Quality evaluation of the articles according to the Jadad scale (Jadad et al., 1996)

	Randomized^a^	Double blind^a^	Lost to follow up and removed from study^a^	Correct method of randomization^b^	Correct method of double blind?^b^	Score
Awad, Lund, Shapiro, et al., 2003	1	0	1	1	−1	**2**
Awad, Lund, Dufresne, et al., 2003	1	0	1	1	−1	**2**
Heydecke et al., 2003	1	0	1	1	−1	**2**
Meijer et al., 2003	1	0	1	1	−1	**2**
Raghoebar et al., 2003	1	0	1	−1	−1	**0**
Thomason et al., 2003	1	0	1	1	−1	**2**
Pan et al., 2008	1	0	1	1	−1	**2**
Harris et al. 2013	1	0	1	1	−1	**2**

^a^ Yes: 1 point/No: 0 points.

^b^ Yes: 1 point/No: 0 points.

The total population included was of 812 patients, 400 of the patients belonged to the CCD group and the other 412 patients belonged to the IOD group. The medium age of the patients was of 64.3 ± 6.41 years. The population was comprised of 283 men and 427 women; despite having article by Awad, Lund, Dufresne, et al. (2003), where the sex was not specified. The retention method for the prosthesis was the bar or ball system, with 285 and 527 patients respectively (Tables 2 and 3).

**TABLE 2:** Characteristics of the reviewed articles

Author	N/Retention type	Age	Sex	Treatment type	RD	TG	Validation instruments	Follow‐up
Awad, Lund, Shapiro, et al., 2003	60 balls	69.3 ± 3.1	25 M	New CCD s/i	Yes	30 CCD 30 IOD	VAS OHIP‐49 OHIP‐Edent	2 m
			35 W	New CCDs/IODi				
Awad, Lund, Dufresne, et al., 2003	102 bar	50.3 ± 6.5	‐	New CCD s/i	Yes	48 CCD 54 IOD	Proper validated questionnaire. VAS y escala Likert	2 m
				New CCDs/IODi				
Heydecke et al., 2003	55 balls	69.4 ± 2.7 DDC	24 M	New CCD s/i	Yes	25 CCD 30 IOD	OHIP‐20 Questionnaire SF‐36 (VAS)	6 m
		68.9 ± 3.2 IOD	31 W	New CCDs/IODi				
Meijer et al., 2003	121 bar	57.8 ± 10.9 DDC	40 M	New CCD s/i	Yes	60 CCD 61 IOD	Proper validated questionnaire. Likert scale and VAS	10 y
		56.9 ± 11.6 IOD	81 W	New CCDs/IODi				
Raghoebar et al., 2003	62 bar	55.2 ± 11.6 DDC	28 M	New CCD s/i	Yes	30 CCD 32 IOD	Likert scale and VAS	10 y
		58.2 ± 12.6 IOD	34 W	New CCDs/IODi				
Thomason et al., 2003	60 balls	70.8 ± 3 DDC	24 M	New CCD s/i	Yes	30 CCD 30 IOD	Proper validated questionnaire. VAS	6 m
		70.1 ± 3.2 IOD	36 W	New CCDs/IODi				
Pan et al., 2008	230 balls	72.3 ± 4.6	103 M	New CCD s/i	Yes	117 CCD 113 IOD	The McGill denture satisfaction instrument (VAS)	12 m
			127 W	New CCD s/IODi				
Harris et al., 2013	122 balls	64.4 ± 7.8	39 M	New CCD s/i	Yes	60 CCD/IOD 62 CCD	CSP (VAS) OHIP‐49	3 m
			83 W	New CCD s/IODi				

*Note*: Age is presented as mean ± SD.

Abbreviations: CCD, Conventional complete denture; CSP, cuestionariode satisfacción de la prótesis; IOD, Implant retained overdenture; M, Male; m, months; OHIP, Oral Health Impact Profile; OHIP‐EDENT, Oral Health Impact profile in edentulous patients; RD, Reference Data (baseline); s/i, superior/ inferior; TG, Treatment group; VAS, Visual Analog Scale; W, Woman; y, years.

**TABLE 3:** Table of the most significant results

Author/Year	Most significant results
Awad, Lund, Shapiro, et al., 2003	*Own questionnaire*: ‐The overall satisfaction was significantly greater in the implant group. ‐The overall satisfaction, the comfort stability and the chewing improvement by the prosthesis is significantly better for the implant group. ‐There are no significant differences between both groups in reference to aesthetics, hygiene or speech.
Awad, Lund, Dufresne, et al., 2003	*Own questionnaire*, *OHIP‐49*, *OHIP‐EDENT*: ‐The overall satisfaction is greater in the implant group. The comfort, stability and the overall ability to chew are significantly better in the implant group. ‐The hygiene easiness in the implant group decreased. ‐There was a general post treatment improvement in both groups. ‐Between the groups, there were only significant differences in the physical pain. ‐In the post treatment the implant group had a significantly lower score. ‐In comparison with the start, the CCD group, significantly reduced the score; as well as in the categories of functional limitations and psychological discomfort.
Heydecke et al., 2003	*OHIP‐20, SF‐36*: ‐At the start there were no differences between groups. ‐Inside both groups, in the CCD group within the subscales of the OHIP of physical pain and psychological discomfort the scores were significantly lower at 6 months. In the IOD group a significant improvement was found within all subscales. ‐Comparing both groups, the total score of the OHIP‐20 is significantly lower in the IOD group. With regards to the OHIP categories, the IOD group has significantly lower scores in four categories: Functional limitation, physical pain, physical inability and psychological inability.
Meijer et al., 2003	*Own questionnaire*: ‐At the start of the study there were no significant differences between both groups. −1 year after treatment the IOD group reported significantly better results than the CCD group with regards to all items, except for the functional complaints of the maxilar CCD in both groups. ‐At 5 years the significant differences between both groups remained the same with regards to all items, except for the functional complaints of the maxilar CCD in both groups. ‐At 10 years the significant differences between groups with regards to the complaints about the mandibular CCD and the satisfaction score, not so with the items about the maxillary CCD and chewing.
Raghoebar et al., 2003	*Own questionnaire*: Significant improvement in the IOD group. At 10 years 40% of the patients that belonged to the non‐implant groups ended up receiving implant treatment.
Thomason et al., 2003	*Own questionnaire*: ‐The satisfaction in the majority of the variables increased significantly, except in the easiness of the hygiene of the prosthesis, in both groups with regards to the initial situation. ‐The overall satisfaction is greater in the implant group. The stability, comfort and the chewing ability is significantly greater in the implant group. ‐There are no significant differences in the chewing ability of certain foods (sausage, bread and lettuce), the aesthetics and the easiness of hygiene are worse in the IOD.
Pan et al., 2008	*Own questionnaire*: ‐The comparison between both treatments presents statistical significant differences after the treatment. ‐The IOD group presents a significantly higher score in overall satisfaction, comfort, stability, chewing capacity and aesthetics. ‐No significant differences between the comparison at 6 and 12 months within the same group in both groups. ‐At the start, women presented a significantly lower score in aesthetics and the chewing capability than men.
Harris et al., 2013	*OHIP, PSQ*: Significant improvement in the IOD group in all subscales of the OHIP‐49. IOD group presents a significant improvement in all the categories of the PSQ except in the rejection of social activities due to problems with the IOD.

Abbreviations: CCD, Conventional complete dentures; IOD, Implant retained overdentures; OHIP, Oral Health Impact Profile; OHIP‐EDENT, Oral Health Impact profile in edentulous patients; OHRQoL, Oral Health Related Quality of Life; PSQ, Prosthesis satisfaction questionnaire; VAS, Analog Visual Scale.

After analyzing the different parameters from the articles that took them into consideration we present:

i. Satisfaction

For the analysis of the satisfaction we ruled out one article (Heydecke et al., 2003) since it did not present a specific category of satisfaction, and it analyzed it with other parameters.

**FIGURE 2:**
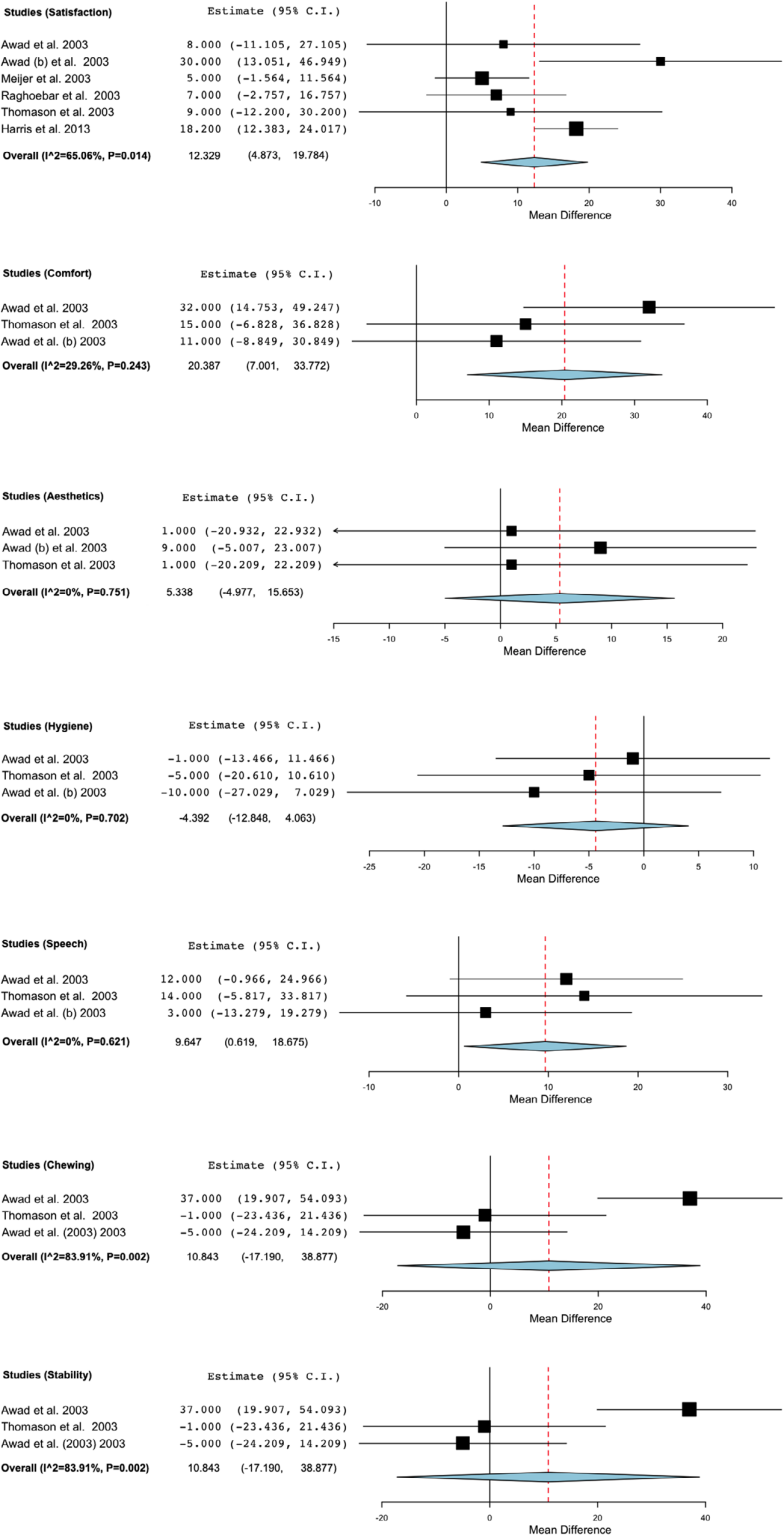
Forest plot assessing the difference in the visual analog scale (VAS) scale pre and post treatment

To study the difference between pre and post treatment, of the seven remaining articles we discarded Pan et al., 2008 study, that only provided data of the post treatment. A total of six RCTs (Awad, Lund, Dufresne, et al., 2003; Awad, Lund, Shapiro, et al., 2003; Harris et al., 2013; Meijer et al., 2003; Raghoebar et al., 2003; Thomason et al., 2003) remain, with a total of 527 patients that were included into the analysis. The difference between the pre and post treatment was statistically significant in favor of the IOD group (Weighted Mean Difference (WMD):12.329; 95% CI: 4.873 to 19.784, *p* = 0.001 and heterogeneity I^2:65.06%, Q(df = 5): 14.31, *p* = 0.014) (Figure 2).

**FIGURE 3:**
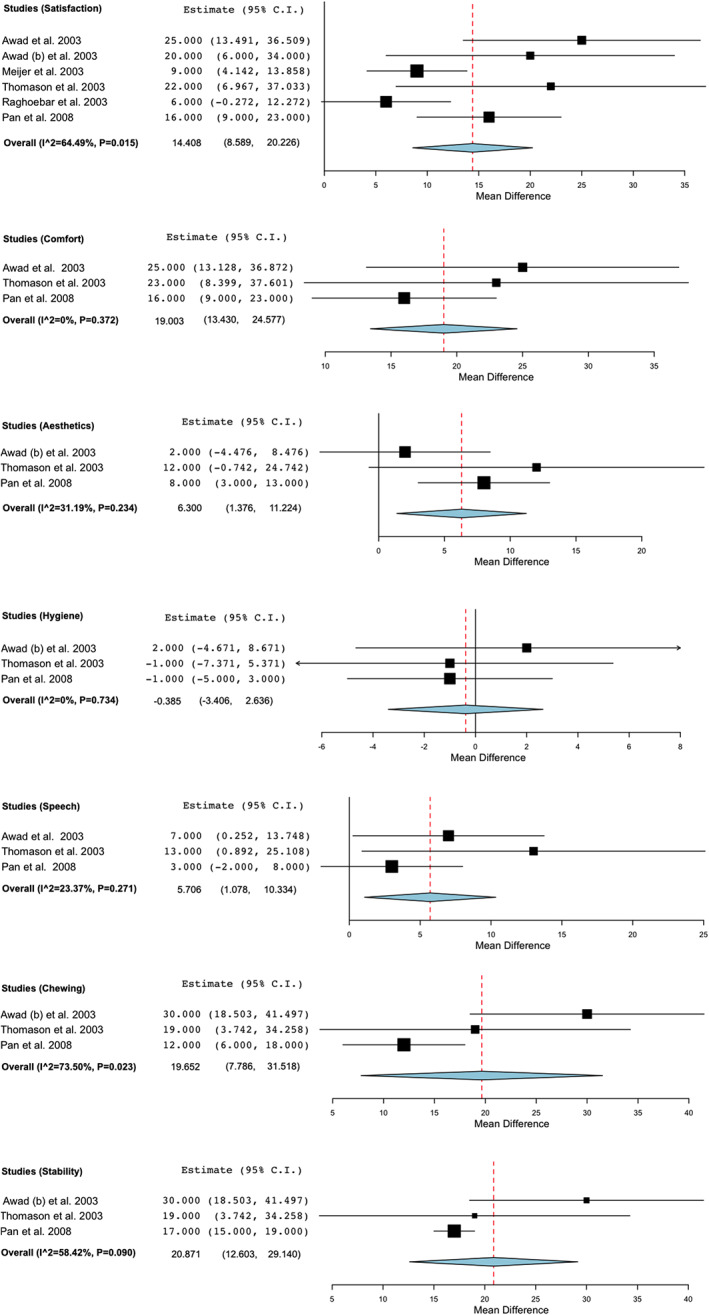
Forest plot assessing the differences in visual analog scale (VAS) scale post treatment

If we concentrate on the final result of the meta‐analysis (Figure 3), we not only discard the article from Heydecke et al. (2003) we also discard the article from Harris et al. (2013) given we only know the information on the difference between the initial status before the treatment and the one after the treatment. Finally, we included six RCT with 635 patients (Awad, Lund, Dufresne, et al., 2003; Awad, Lund, Shapiro, et al., 2003; Meijer et al., 2003; Pan et al., 2008; Raghoebar et al., 2003; Thomason et al., 2003), where a statistically significant difference was observed in favor of the IOD group (WMD: 14.408; 95% CI:8.589 to 20.226, *p* < 0.001 and heterogeneity I^2:64.492%, Q(df = 5): 14.081, *p* = 0.015).

The categories of comfort, aesthetics, hygiene, speech, chewing and stability were analyzed by four studies (Awad, Lund, Dufresne, et al., 2003; Awad, Lund, Shapiro, et al., 2003; Pan et al., 2008; Thomason et al., 2003). In order to obtain in the meta‐analysis the difference between both modalities of treatment before and after we discarded the study of Pan et al. (2008) since it only provided data of the post treatment.

In our meta‐analysis in order to attain the value of the end result between groups we discarded the study of Awad, Lund, Shapiro, et al. (2003) since it did not provide information in the VAS scale of the end treatment results, it only provided data of the difference before and after the treatment.

ii. Comfort

From a total of 222 patients belonging to 3 RCT (Awad, Lund, Dufresne, et al., 2003; Awad, Lund, Shapiro, et al., 2003; Thomason et al., 2003) included in the meta‐analysis (Figure 2), a statistically significant difference in favor of the IOD group was observed (WMD: 20.387; 95% CI: 7.001 to 33.772, *p* = 0.003 and heterogeneity I^2:29.26%, Q(df = 2): 2.827, *p* = 0.243). Basing ourselves only in the final result 392 patients of three RCT (Awad, Lund, Dufresne, et al., 2003; Pan et al., 2008; Thomason et al., 2003) were presented (Figure 3), where the difference was statistically significant in favor of the treatment with IOD (WMD: 19.003; 95% CI: 13.420 to 24.577, *p* < 0.001 and heterogeneity I^2:0%, Q(df = 2): 1.975, *p* = 0.372).

iii. Aesthetics

222 patients studied in three RCT (Awad, Lund, Dufresne, et al., 2003; Awad, Lund, Shapiro, et al., 2003; Thomason et al., 2003) were included (Figure 2). The difference was not statistically significant when comparing between the CCD and IOD groups pre and post treatment results (WMD: 5.338; 95% CI: −4.997 to 15.653, *p* = 0.310 and heterogeneity I^2:0%, Q(df = 2):0.574, *p* = 0.751). Analyzing only the final results between both treatments on patients of the 3 RCT (Awad, Lund, Dufresne, et al., 2003; Pan et al., 2008; Thomason et al., 2003) (Figure 3) that includes a total of 392 patients, the difference was statistically significant in favor of the IOD group (WMD: 6.3; 95% CI: 1.376 to 11.224, *p* = 0.046 and heterogeneity I^2:31.188%, Q(df = 2):2.906, *p* = 0.234).

iv. Hygiene

From a total of RCT (Awad, Lund, Dufresne, et al., 2003; Awad, Lund, Shapiro, et al., 2003; Thomason et al., 2003) 222 patients were analyzed (Figure 2). The differences between the initial situation and the post treatment of the two groups were not statistically significant, even though the hygiene worsened since the initial situation (WMD: ‐4.392; 95% CI: −12.848 to 4.063, *p* = 0.309 and heterogeneity I^2:0%, Q(df = 2):0.707, *p* = 0.702). Taking into account only the final situation after performing the treatment, we analyzed 392 patients of three RCT (Awad, Lund, Dufresne, et al., 2003; Pan et al., 2008; Thomason et al., 2003) (Figure 3), the difference was not statistically significant even though the situation worsened in the IOD group (WMD: ‐0.385; 95% CI: −3.406 to 2.636, *p* = 0.855 and heterogeneity I^2 = 0%, Q(df = 2): 0.618, *p* = 0.734).

v. Speech

For the meta‐analysis 222 patients belonging to three RCT (Awad, Lund, Shapiro, et al., 2003; Thomason et al., 2003; Yamamoto & Shiga, 2018) were included (Figure 2). Basing ourselves in the difference between the state before and after the treatment we found that there was a statistically significant improvement in favor of the IOD group (WMD: 9.647; 95% CI: 0.619 to 18.675, *p* = 0.036 and heterogeneity I^2 = 0%, Q(df = 2):0.952, *p* = 0.621). 3 RCT (Awad, Lund, Dufresne, et al., 2003; Pan et al., 2008; Thomason et al., 2003) with 392 patients were analyzed (Figure 3) in relation to the comparison between the final state of both treatments groups, this results in a statistically significant improvement in favor of the IOD group (WMD: 5.706; 95% CI: 1.078 to 10.334, *p* = 0.016 and heterogeneity I^2 = 23.37%, Q(df = 2): 2.724, *p* = 0.271).

vi. Chewing

222 patients from three RCT (Awad, Lund, Dufresne, et al., 2003; Awad, Lund, Shapiro, et al., 2003; Thomason et al., 2003) (Figure 2) were studied to compare the difference between the results pre and post treatment of both groups, where no statistically significant difference was found, even though it favored slightly the IOD group (WMD: 10.843; 95% CI: −17.190 to 38.887, *p* = 0.448 and heterogeneity I^2 = 83.91%, Q(df = 2):12.427, *p* = 0.002). The results of three RCT (Awad, Lund, Dufresne, et al., 2003; Pan et al., 2008; Thomason et al., 2003) with 392 patients can be analyzed in regards to the final treatment difference between both groups, which was statistically significant as well in favor of the IOD group (WMD: 19.652; 95%, CI: 7.786 to 31.518, *p* = 0.001 and heterogeneity I^2:73.50%, Q(df = 2): 7.547, *p* = 0.023) (Figure 3).

vii. Stability

The stability was analyzed in 222 patients of three RCT (Awad, Lund, Dufresne, et al., 2003; Awad, Lund, Shapiro, et al., 2003; Thomason et al., 2003) (Figure 2). Taking into account the overall change over the initial situation and the final situation of the treatment the difference was statistically significant in favor of the IOD group (WMD: 23.871; 95% CI:11.776 to 35.966, *p* < 0.001 and heterogeneity I^2 = 22.72%, Q(df = 2):2.588, *p* = 0.274). The stability at the end of the treatment was compared among 392 patients belonging to three RCT (Awad, Lund, Dufresne, et al., 2003; Pan et al., 2008; Thomason et al., 2003) (Figure 3), with a statistically significant difference in favor of the IOD group as well (WMD: 20.871; 95% CI: 12.603 to 29.140, *p* < 0.001 and heterogeneity I^2:58.42%, Q(df = 2):4.810, *p* = 0.090).

## 4 DISCUSSION

In this systematic review and meta‐analysis, the satisfaction and quality of life of patients treated with mandibular IOD and CCD with opposing maxillary CCD is analyzed. The results obtained demonstrated that the treatment with mandibular implants is more effective according to the patient satisfaction index or Oral Health Related Quality of Life (OHRQoL) (Allen et al., 2006; Harris et al., 2013; Heydecke et al., 2005; Meijer et al., 2003; Thomason et al., 2003; Visser et al., 2006).

In our work we performed two comparisons; in one we compared the treatment final result valuations between both groups and in the other we analyzed the differences in the scores between the pre and post treatment groups. After reviewing the meta‐analysis, we saw that the overall satisfaction, as well as the majority of subcategories, was statistically significant in favor of the IOD group. The only category that was not statistically significant or even worse was the hygiene in the IOD treatment group, taking into account that these IOD prostheses have more structures that require more cleaning than CCD.

Furthermore, these prostheses are usually done on elderly patients who have a lesser cognitive and manual capability, which can worsen the hygienic maintenance with the passing of time (Y. Zhang et al., 2019). According to data provided by authors such as Al‐Magaleh et al. (2017) they assure that IOD over 4 implants have no statistically significant differences in comparison with structures over 2 implants; so by performing these treatments we facilitate the hygiene without sacrificing quality of life or the satisfaction of those older patients with less manual skill that have difficulties to perform the hygiene of their prostheses.

With regards to chewing, it came up to our attention, when analyzing the difference between the pre and post treatment between both groups this was not statistically significant. This means that only with a new well‐adjusted prosthesis patient already perceive an improvement in their chewing. However, if we only focus in the final result, we can observe that the patients with IOD perceived an improvement in functionality (statistically significant) over the patients that used CCD. This result is in agreement with the one found by the study of Yamamoto and Shiga (2018) in which they provided new CCD and a significant improvement in the masticatory perception and the quality of life was noted.

The obtained results are bolstered by the work of Sivaramakrishnan and Sridharan (2016). These authors carried out in 2016 a meta‐analysis of RCTs where they analyzed the quality of life through OHIP questionnaires. The OHIP questionnaires, with all their variables (OHIP‐49, OHIP‐14, OHIP‐EDENT), are a tool to evaluate the OHRQoL, where a lower score implies a better quality of life (Slade & Spencer, 1994). These questionnaires show statistically significant differences in favor of the IOD group when compared with the CCD group (lesser score in the IOD group). These results coincide with the ones in our study where the VAS scale was used (which allows us to obtain a more subjective opinion of the patients' experience). The investigators propose that the first option for the rehabilitation of edentulous patients must be the IOD. More so, in those patients with severe bone resorption, given they provide more stability and better capacity for chewing (Sivaramakrishnan & Sridharan, 2016). In contrast with this study, in our work we used VAS type scales that allowed us to obtain much more subjective information of the patients' experience.

In reference to the number of necessary implants, there are several articles that studied the differences with regards to the satisfaction of the patients. Some have concluded that the difference between two and four implants is not statistically significant (Al‐Magaleh et al., 2017; Lee et al., 2012); furthermore we must take into account other factors such as the cost of the treatment (L. Zhang et al., 2017), providing a treatment with les morbidity to the patient or, as we already addressed, with an easier maintenance (Y. Zhang et al., 2019).

One of the strong points of this review is the inclusion of solely RCT and results based on the experience of the patient. The subjective results in this kind of analyses are considered appropriate, given that the finality of the treatment is oriented toward improving the function, satisfaction and the patient's quality of life, in an effective manner, thus the patient's perspective has to be taken into account (Kriz et al., 2012). Nonetheless, authors such as Kroll et al. (2018) propose that future investigation should concentrate as well in the objective results obtained, such as the chewing efficiency, communication tests and the muscular coordination through electromyography.

Some limitations present within this meta‐analysis exist due to the disparity of studies that analyze the different aspects of the satisfaction and quality of life. We also found that the number of RCTs is limited, of low quality and the studies that we selected represent moderate heterogeneity measured with I^2 and Cochran's Q test Despite these studies being the *gold standard* they can also present biases that will affect the quality and value of the study. For example, no study was double blind (since this would be hard to implement given the nature of the study). Another limitation is the heterogeneity that the studies present and their short follow‐up period.

## 5 CONCLUSIONS

The results of this systematic review and meta‐analysis indicate that the mandibular IOD over two implants have a better subjective evaluation than the CCD. Overall the satisfaction was better with regards to comfort, speech, stability and chewing; there was no significant improvement in the aesthetics and a worse satisfaction with the hygiene.

The RCT studies analyzed present a short follow‐up period, so future investigations should consider having longer follow‐up periods to evaluate the consistency of the results.

## CONFLICT OF INTEREST

The authors did not report any conflict of interest.

## AUTHOR CONTRIBUTIONS

*Initial research*: Sonia Egido Moreno, Mayra Schemel Suárez, Joan Valls Roca‐Umbert and Keila Izquierdo Gómez. *Articles were reviewed*: Raúl Ayuso Montero. *Any disagreements were settled*: José López López.
